# Reply to the Comment on “The m6A Reader IGF2BP2 Regulates Macrophage Phenotypic Activation and Inflammatory Diseases by Stabilizing TSC1 and PPAR*γ*”

**DOI:** 10.1002/advs.202201452

**Published:** 2022-07-12

**Authors:** Xia Wang, Shuai Xu, Dawei Chen

**Affiliations:** ^1^ Laboratory of Translational Gastroenterology Department of Gastroenterology Qilu Hospital Shandong University Jinan 250012 China; ^2^ Department of Obstetrics and Gynecology The Second Hospital Cheeloo College of Medicine Shandong University Jinan 250033 China; ^3^ Laboratory of Medical Chemistry Interdisciplinary Cluster for Applied Genoproteomics GIGA‐Stem Cells Faculty of Medicine University of Liège CHU, Sart‐Tilman Liège 4000 Belgium

**Keywords:** IGF2BP2, macrophage polarization

We thank Schymik et al.^[^ ^1^ ^]^ for their interest in our article published in the July 2021 issue.^[^ ^2^ ^]^ They raised several interesting points that we would like to address.

First, the concern “in human monocyte‐derived macrophages (HMDMs) treated with bacterial lipopolysaccharide (LPS) for up to 24 h, result in a significant reduction in insulin‐like growth factor 2 mRNA‐binding protein 2 (IGF2BP2) mRNA expression, while protein levels were not changed” is a very interesting consideration, which is different from what we claimed in the mouse bone marrow‐derived macrophages (BMDMs). In order to understand why LPS regulates macrophage polarization differently between murine and human, we first performed similar experiments with what Schymik et al. did.^[^ ^1^ ^]^ Short‐time LPS treatment slightly induced the expression of IGF2BP2 mRNA (**Figure** **1**A), while IGF2BP2 protein levels did not change (Figure 1B,C). Interestingly, the mRNA (Figure 1A) and protein (Figure 1D,E) levels of IGF2BP2 were significantly enhanced by LPS treatment for 36 and 48 h, which is consistent with mouse BMDMs.

**Figure 1:**
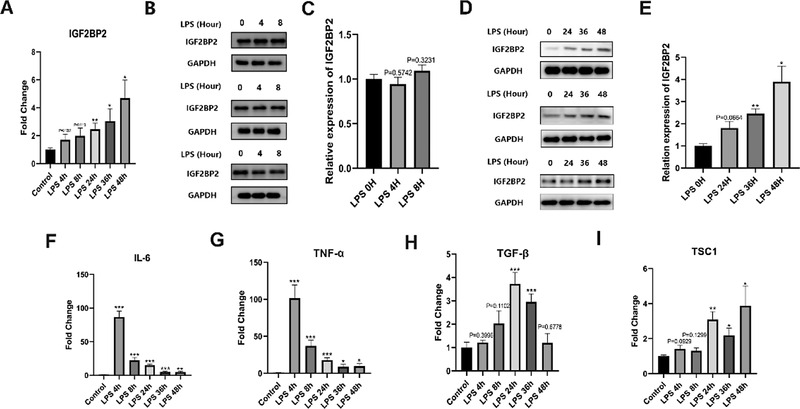
IGF2BP2 expression in LPS activated human macrophages. A) IGF2BP2 mRNA level in HMDMs treated by saline (Control) or LPS (100 ng mL^−1^) at indicated time. B) IGF2BP2 protein expression in HMDMs treated by normal saline or LPS (100 ng mL^−1^) for 4 and 8 h. C) Statistically analysis of (B). D) IGF2BP2 protein expression in HMDMs treated by saline or LPS (100 ng mL^−1^) for 24, 36, and 48 h. E) Statistically analysis of (D). F–I) Inflammatory cytokine and TSC1 mRNA level in HMDMs determined by RT‐qPCR after normal saline or LPS treatment at indicated time points; qPCR biological replicates come from six different donors; western blot biological replicates come from three different donors; anti‐IGF2BP2 antibody was purchased from Proteintech (11601‐1‐AP). The mRNA levels in the Control group were set to 1 and levels in others experimental conditions were relative to that after normalization with GAPDH. The dates were shown as mean ± SEM. *p < 0.05, **p < 0.01, ***p < 0.001, versus the control group. *p*‐values were determined by using two‐way analysis of variance (ANOVA).

Notably, the plastic adhesion method was used instead of magnetic bead sorting to extract human monocytes, which might cause different phenotypes of HMDMs^[^ ^3^ ^]^ by LPS treatment we got compared to Schymik et al. To exploit whether isolation and differentiation procedures result in a different activation profile, we also measured the expression of inflammation‐associated genes. At early M1 activation time points, obvious up‐regulation of inflammatory cytokines Interleukin‐6 (IL‐6) and tumor necrosis factor *α* (TNF‐*α*) was observed (Figure 1F,G), which is similar to Schymik et al's. Importantly, the reduction of the IL6 and TNF‐*α* (Figure 1F,G) and augment of the anti‐inflammatory cytokine transforming growth factor‐beta (TGF‐*β*) (Figure 1H) was accompanied by the induction of IGF2BP2 mRNA (Figure 1A–E). Meanwhile, in line with the tendency of IGF2BP2, the expression of Tuberous sclerosis 1 (TSC1), which was mediated by IGF2BP2, was distinctly elevated upon LPS treatment (Figure 1I). These data indicate that IGF2BP2 increased during the resolution of inflammation in HMDMs.

Additionally, compared with HMDMs, the expression of IGF2BP2 apparently increased in short‐term LPS stimulated BMDMs (**Figure** **2**), which may be due to different toll‐like receptor regulated gene expression in primary human and mouse macrophages.^[^ ^4^ ^]^ Additionally, accumulating evidence indicates that the shift of metabolic reprogramming is a hallmark of activated inflammatory (M1) macrophages.^[^ ^5^, ^6^ ^]^ We previously reported that IGF2BP2 regulates the murine macrophage polarization via metabolic reprogramming,^[^ ^2^ ^]^ however, human and murine macrophages exhibit differential metabolic responses to LPS,^[^ ^7^ ^]^ which could also cause the different IGF2BP2 expression patterns between HMDMs and BMDMs.

**Figure 2:**
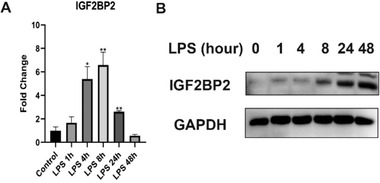
IGF2BP2 expression in LPS activated mouse macrophages. A) IGF2BP2 mRNA level in BMDMs treated by saline (Control) or LPS (100 ng mL^−1^) at the indicated time. B) IGF2BP2 protein expression in BMDMs treated by normal saline or LPS (100 ng mL^−1^) at the indicated time points, anti‐IGF2BP2 antibody was purchased from Proteintech (11601‐1‐AP); qPCR and western blot biological replicates from three different donors; IGF2BP2 mRNA levels in Control group were set to 1 and levels in others experimental conditions were relative to that after normalization with GAPDH. The dates were shown as mean ± SEM. *p < 0.05, **p < 0.01, versus the Control group. *p*‐values were determined by using a two‐way analysis of variance (ANOVA).

Finally, the data of M2 polarized human macrophages offered by Schymik et al.^[^ ^1^ ^]^ supported that Interleukin‐4 (IL‐4) can promote IGF2BP2 expression in both human and mouse macrophages, which implies that the induction of IGF2BP2 expression by IL‐4 via STAT6/HMGA2 pathway^[^ ^2^ ^]^ is conserved among species. Thus, targeting IGF2BP2 in M2 macrophages may be a potential therapy to treat asthma or cancer.

Overall, the findings of the N6‐methyladenosine reader protein IGF2BP2 function in mouse macrophage polarization will potentially provide a drug target to regulate macrophage activation, which will be beneficial to M2‐governed inflammatory diseases treatments for human beings. Here, we also confirmed that long‐time treatment with LPS increased IGF2BP2 expression of both mRNA and protein levels in HMDMs. Further studies are needed to understand how Toll‐like receprtors (TLR) agonists and metabolic reprogramming regulate IGF2BP2 expression in both murine and human macrophages.

## 1 Experimental Section

### HMDM Isolation and Differentiation

HMDMs were isolated and differentiated as described before.^[^ ^3^ ^]^ Blood samples of women aged around 30 years old were obtained from The Second Hospital of Shandong University. Permission to use human materials for primary cell isolation was approved by the local ethics committee (KYLL‐2019 (KJ) P‐0128).

Peripheral blood mononuclear cells (PBMCs) were isolated by density gradient centrifugation using Lymphocyte Separation Medium 1077 (PromoCell, Heidelberg, Germany, #C‐44010). PBMCs were washed once in phosphate‐buffered saline (PBS) with 2% fetal calf serum (FCS) and resuspended in RPMI‐1640 (ThermoFisher Scientific) with 10% human AB serum (Sigma‐Aldrich). For monocyte isolation, 1 × 10^8^ to 2 × 10^8^ PBMCs were plated in Nuclon Delta surface‐treated T‐75 cell culture flasks (ThermoFisher Scientific) and allowed to adhere in a 5% CO_2_ container at 37 °C for 1 h. Nonadherent cells were removed by washing with RPMI‐1640. Adherent cells were harvested and cultured in nontreated T‐75 flasks with complete maturation media (RPMI‐1640, 10% FCS, 100 U/100 µg mL^−1^ penicillin/streptomycin (ThermoFisher Scientific), 10 ng mL^−1^ macrophage colony‐stimulating factor (M‐CSF) (Peprotech, Stockholm, Sweden) for 6 days. Media were changed every 2–3 days.

### RNA Extraction and Real‐Time PCR Analysis

The level of mRNA expressions was defined by reverse transcription‐polymerase chain reaction and RT‐qPCR. RNA was gained from cells with EASYspin Plus kit (Aidlab) and then synthesized to QuantiTect RevComplementary DNA (cDNA) by using the QuantiTect Rev. Transcription Kit (Vazyme, Nanjing) and augmented by using SYBR Green qPCR Mix (Vazyme, Nanjing) on. △△Ct values were normalized to GAPDH, and relative quantification of gene expression was compared to the Control group. Bioer–Lightcycler The primers used in this study are synthesized by the Beijing Genomics Institute (Beijing, China).

Primer Sequence

human_GAPDH_fw GGAGCGAGATCCCTCCAAAAT

human_GAPDH_rev GGCTGTTGTCATACTTCTCATGG

human_IGF2BP2_fw GTTCCCGCATCATCACTCTTAT

human_IGF2BP2_rev GAATCTCGCCAGCTGTTTGA

human_IL‐6_fw ACATCCTCGACGGCATCTCA

human_IL‐6_rev TCACCAGGCAAGTCTCCTCATT

human_TGF‐*β*_fw GTGGACATCAACGGGTTCACT

human_TGF‐*β*_rev CGCACGCAGCAGTTCTTCTC

human_TNF‐*α*_fw CTCCACCCATGTGCTCCTCA

human_TNF‐*α*_rev CTCTGGCAGGGGCTCTTGAT

human_TSC1_fw AGAGCCACATGACAAGCACC

human_TSC1_rev GGATAAACGAGTGGCGGCTT

mouse_GAPDH_fw AGGTCGGTGTGAACGGATTTG

mouse_GAPDH_rev TGTAGACCATGTAGTTGAGGTCA

mouse_IGF2BP2_fw GTCCTACTCAAGTCCGGCTAC

mouse_IGF2BP2_rev CATATTCAGCCAACAGCCCAT

## Conflict of Interest

The authors declare no conflict of interest.

## Data Availability

Research data are not shared.
